# Exploring Acute Pancreatitis in Kidney Transplant Recipients: A Multicentre Retrospective Cohort Analysis of Incidence, Causes, and Clinical Outcomes

**DOI:** 10.3390/jcm13123366

**Published:** 2024-06-07

**Authors:** Nikolina Basic-Jukic, Alen Androvic, David Beck, Danilo Radunovic, Ivana Juric, Vesna Furic-Cunko, Lea Katalinic, Zoran Sabljic, Margareta Fistrek-Prlic, Armin Atic, Marina Kljajic, Bojan Jelakovic

**Affiliations:** 1 Department of Nephrology, Arterial Hypertension, Dialysis and Transplantation, University Hospital Centre Zagreb, 10000 Zagreb, Croatia; 2 School of Medicine, University of Zagreb, 10000 Zagreb, Croatia; 3 Department of Nephrology, General Hospital Varazdin, 42000 Varaždin, Croatia; 4 Department of Nephrology, Clinical Center Montenegro, 81000 Podgorica, Montenegro

**Keywords:** acute pancreatitis, kidney transplantation, sirolimus, tacrolimus, cannabis

## Abstract

**Background**: The aim of this multicentre retrospective study is to determine the incidence, etiology, clinical characteristics, and outcomes of kidney transplant recipients diagnosed and treated for acute pancreatitis. **Methods**: We analyzed data from kidney transplant recipients who received kidney allografts between October 1973 and December 2023 and were diagnosed and treated for acute pancreatitis. **Results**: Of 2482 patients who received kidney allografts, 10 (0.4%) (5 male) were diagnosed with acute pancreatitis, with a mean age of 48.6 years. Patients were diagnosed with acute pancreatitis between 3 weeks and 24 years after the transplantation. Possible etiologies included cholecystolithiasis, COVID-19, hypercalcemia, postprocedural, use of cannabis, trimetoprim-sulphometoxasole, statins, sirolimus, tacrolimus and obesity. There was no suspected etiology in two patients. Patients were treated with aggressive hydration, pain alleviation and antibiotics if indicated. Four patients developed complications. Local complications included peripancreatic collections, pseudocyst, and abscesses formation, while systemic complications occurred in the form of Cytomegalovirus (CMV) reactivation and urinary tract infection. All patients survived with preserved kidney allograft function. **Conclusions**: Acute pancreatitis in kidney transplant recipients is rare. However, it may be linked to significant morbidity and mortality. While symptoms may be nonspecific and brought on by a variety of viral and non-infectious illnesses, as well as adverse effects from immunosuppressive medications, a high degree of awareness is required.

## 1. Introduction

Gastrointestinal complications are among the leading causes of morbidity and mortality in immunocompromised patients [1], with up to 92% of renal transplant recipients developing gastrointestinal symptoms ranging in severity from mild diarrhea to life-threatening problems [2,3,4]. Numerous coexisting pathologies, which include drug toxicity, pathoanatomic processes, diabetes, acidosis and metabolic syndrome, aggravate the determination of the etiology of gastrointestinal problems. Additionally, the clinical signs and symptoms of gastrointestinal complications arising after kidney transplantation may be altered by the immunosuppressive drugs [1,5]. Thus, although frequent, gastrointestinal symptoms and complications are often neglected by nephrologists [4]. Acute pancreatitis is a common disease in the general population, which is associated with significant morbidity and mortality. The incidence of acute pancreatitis varies between 3.4 and 73.4 cases per 100,000 worldwide and has been increasing by 2–5% per year [6,7,8]. The severity of the disease ranges from mild abdominal discomfort to a life-threatening condition. In its most severe form, acute pancreatitis promotes a systemic inflammatory response with multiple organ failure and an increased risk of infective complications. Mortality rates at this stage may approach 30% [9]. Although most of the patients who experience acute pancreatitis survive the acute disease, many of them may suffer long-term consequences, including chronic pancreatitis, recurrent episodes of acute pancreatitis [10], and pancreatic insufficiency (exocrine and endocrine) [11]. Additionally, acute pancreatitis is associated with an increased risk of pancreatic cancer [12]. Treatment options for acute pancreatitis are still limited to supportive therapy and are based on aggressive fluid resuscitation [13,14], pain alleviation, and nutritional support [15]. Currently, no specific therapies are approved for the treatment of acute pancreatitis. Data regarding acute pancreatitis in kidney transplant recipients are limited. This multicentre, multinational, retrospective observational cohort study aimed to describe the clinical characteristics and outcomes of patients with acute pancreatitis after kidney transplantation.

## 2. Materials and Methods

Patients with acute pancreatitis or acutization of chronic pancreatitis diagnosed between October 1973 and December 2023 at University Hospital Centre Zagreb (Croatia), General Hospital Varazdin (Croatia) and Clinical Hospital Montenegro (Montenegro) were included in this multicentre, retrospective cohort study. Data were obtained from medical charts and records. The diagnosis of acute pancreatitis was established according to the revised Atlanta classification by identifying 2 of the following 3 criteria: (1) abdominal pain consistent with the disease, (2) serum amylase and/or lipase greater than 3 times the upper limit of normal, and/or (3) characteristic findings from abdominal imaging [16]. Severity was determined according to the aforementioned classification, with the presence of persistent organ failure longer than 48 h and/or death indicating severe acute pancreatitis. Transient organ failure lasting less than 48 h with/without the development of local complications (acute pancreatic and/or peripancreatic fluid collections, acute necrotic collections, pseudocyst, or walled-off pancreatic necrosis) is defined as moderately severe acute pancreatitis. Organ failure components included in the definition of severe acute pancreatitis are as follows: shock with systolic blood pressure <90 mm Hg PaO_2_ less than 60 mm Hg, indicating respiratory failure, renal failure, and/or gastrointestinal bleeding [16].

Drug-induced acute pancreatitis was defined according to Mallory and Kern: (1) symptoms of acute pancreatitis occur after the administration of the associated medication, (2) clinical symptoms resolve after drug withdrawal, (3) symptoms recur after reintroduction of the suspect drug, and (4) other causes of acute pancreatitis are excluded [17].

Patients were treated with a triple immunosuppressive regimen (calcineurin inhibitor, antiproliferative drug, and steroids). The mammalian target of rapamycin inhibitors was used in patients with malignancies or those with side effects to calcineurin inhibitors. For induction therapy at transplantation, those transplanted from 2009 who were sensitized or were receiving a second or third renal allograft received antithymocyte globulin; others received basiliximab. Since 2009, all patients have received a universal 6-month prophylaxis with valganciclovir and trimethoprim-sulphomethoxazole. 

Age, gender, dialysis vintage, type of dialysis, time of transplantation, immunosuppressive protocol, donor type, acute graft rejection episodes, treatment of rejection episodes, other infections, and graft function were recorded. Detailed clinical characteristics of acute pancreatitis episode(s) were noted. The following parameters were recorded: length of hospital stay, need for surgical intervention, infective complication in any organ system, organ failure requiring support and death.

## 3. Results

### 3.1. Patients’ Characteristics

From October 1973 to December 2023, 2482 patients received kidney allograft at our institutions. Acute pancreatitis was diagnosed in 10 patients (0.4%). There were five male patients and five female patients, with the mean age at diagnosis of 48.6 years (ranging from 24 to 69 years) (Table 1).

Two patients had glomerulonephritis without biopsy, two had unknown disease, two had chronic pyelonephritis, and two had nephroangiosclerosis, while focal segmental glomerulosclerosis, and autosomal dominant polycystic kidney disease were diagnosed in one patient each. 

Patients received kidneys from two living and eight deceased donors. One patient developed acute pancreatitis after the second transplantation. The average time on dialysis was 33.1 months (range 0–104 months). During the pretransplant period, one patient was treated with peritoneal dialysis, five with hemodialysis, and two with both methods. Two patients underwent preemptive kidney transplantation.

### 3.2. Possible Etiology of Acute Pancreatitis

At the time of onset of acute pancreatitis, four patients were treated with cyclosporine, mycophenolate mofetil, and steroids; 5 with tacrolimus, mycophenolate mofetil, and steroids, and one received sirolimus, mycophenolate, and steroid (Table 2). Increased sirolimus trough level and increased tacrolimus through the level at the time of acute pancreatitis occurrence were recorded in one patient each. Mycophenolic acid concentrations were not determined. Nine patients (90%) received induction therapy with either basiliximab (eight patients) or antithymocyte globulin (one patient) in their immunosuppressive protocol. One patient had an episode of acute allograft rejection treated with three methylprednisolone pulses in the early post-transplant period. Three patients received statins at acute pancreatitis (AP) occurrence and three trimethoprim-sulphomethoxasole. Angiotensin-converting enzyme inhibitor (ACEi) was used by one patient.

Three patients developed acute pancreatitis during acute COVID-19. Hypercalcemia and the use of statins were recorded in two patients each. Two patients had cholecystolithiasis. One patient had a cannabis addiction, and one developed acute pancreatitis after the endoscopic retrograde cholangiopancreatography (ERCP). There was no suspected etiology in two patients.

### 3.3. Timing of Acute Pancreatitis

The timing of acute pancreatitis was variable, ranging from 3 weeks to 24 years after the transplantation. Four patients developed acute pancreatitis within the first three months post-transplant. Very late-onset acute pancreatitis, occurring 24 years post-transplant, was caused by cholecystolithiasis.

### 3.4. Clinical Presentation

Abdominal pain was a dominant symptom that occurred in 9 (90%) patients, while five patients had concomitant nausea (50%). Only one patient experienced vomiting within the spectrum of initial symptoms—one patient presented with diarrhea without pain that occurred later in the course of the disease. Radiological imaging was performed on all patients. Computerized tomography revealed parenchymal enlargement in five patients, indistinct pancreatic margins in four patients, and surrounding retroperitoneal fat stranding in seven patients. Two patients had pseudocysts (one patient with additional calcifications in parenchyma), indicating previous episodes of acute pancreatitis. One of them subsequently developed abscesses. One patient had enlarged surrounding lymph nodes (Table 2).

All patients had elevated serum and urine amylases and lipases. Serum triglycerides were within normal range in all patients at the time of acute pancreatitis occurrence. Nine patients had mild acute pancreatitis and one had moderately severe disease requiring surgical intervention and admission to the intensive care unit.

### 3.5. Treatment

All patients were initially treated with aggressive fluid resuscitation. Lactated Ringer solution was used along with analgesic medications and nutritional support. Antibiotics were used in a patient with a *Morganella morganii* urinary tract infection and in a patient with abscess formation. This patient required surgical drainage. Immunosuppressive therapy was decreased in patients with moderately severe acute pancreatitis and in a patient with CMV reactivation. Tacrolimus was switched to cyclosporine in a patient with early post-transplant chronic pancreatitis recurrence. After this conversion, she did not experience additional episodes for two years when a mild episode appeared after the nutritional challenge.

### 3.6. Hospitalization Course, Complications, and Outcome

Most of the patients required 10–14 days of hospital treatment. However, those with complications were hospitalized for longer periods depending on the severity of the disease. Four patients developed complications. Local complications included peripancreatic collections, pseudocyst, and abscess formation, while systemic complications occurred in the form of CMV reactivation and urinary tract infection. All patients survived with preserved kidney allograft function. However, the patient who developed acute pancreatitis after ERCP died one year later from sepsis after a Whipple procedure for the treatment of a mucinous cystic pancreatic tumor.

## 4. Discussion

The current study examined the prevalence of acute pancreatitis, etiology, and outcomes in a multicentre cohort retrospective study. We found a low incidence of acute pancreatitis in kidney transplant recipients (0.4%). Three patients developed acute pancreatitis within the first 3 months post-transplant. Possible etiologic factors included gallstones, immunosuppressive drugs, statins, trimetoprim-sulphomethoxasole, angiotensin convertase inhibitors, cannabis, COVID-19, and hypercalcemia. One patient developed postprocedural acute pancreatitis. Two patients had idiopathic acute pancreatitis. Most of our patients were overweight. Aggressive hydration was the cornerstone of the therapy. Outcomes were satisfactory, with a survival rate of 100%. Two patients had sequelae of acute pancreatitis in the form of pseudocyst formation.

The majority of the literature on acute pancreatitis in kidney transplant recipients is based on case reports, which is surprising given the gravity of the issue. The relative rarity of the disease, presentation masked by the immunosuppressive state, and, possibly, physicians’ underestimation of the problem may all contribute to the scarcity of available data. The etiology of acute pancreatitis most commonly includes gallstones (40–70%) and alcohol consumption (25–35%). Chronic alcohol intake may induce a broad spectrum of pancreatic injury, ranging from mild episodes of acute pancreatitis to chronic irreversible changes. While only approximately 5% of alcoholics develop clinically evident acute pancreatitis, it seems that additional risk factors increase susceptibility to alcohol-induced pancreatic changes [18]. According to Ammann, diagnosis of alcohol-induced acute pancreatitis should be considered in individuals with at least five years of heavy alcohol consumption (more than 50 g of alcohol per day) [19]. Other rare possible etiologies include infections, medications, and metabolic changes (hypertriglyceridemia and hypercalcemia).

Acute pancreatitis was recorded in association with different viral infections (mumps, coxsackie, hepatitis B, cytomegalovirus, varicella-zoster virus, herpes simplex virus), but also with bacterial infections (Salmonella, Mycoplasma, Legionella, and Leptospira), parasitic infections (Toxoplasma, Cryptosporidium, Ascaris) and fungi (Aspergillus) [20]. Some of these infective causes may be especially significant in immunocompromised patients prone to opportunistic infections (20). 

Up to 5% of cases of acute pancreatitis are drug-induced [21], and the most frequently used medications linked to it are atorvastatin, didanosine, azathioprine, and hydrochlorothiazide [22,23]. The possible association of different drugs with the development of acute pancreatitis has been classified into four classes [24]. Drug-induced acute pancreatitis class Ia drugs include medications with at least one case that has a positive rechallenge with the exclusion of other potential causative factors such as alcohol, hypertriglyceridemia, and gallstones. Classified as Ib are drugs with at least one reported case with a positive rechallenge but without the possibility of excluding other potential etiologies of acute pancreatitis. At least four reported cases with coincident latency in more than 75% of cases are required for class II drugs. Class III drugs should include at least two published cases with no consistent latency time and no rechallenge, while class IV drugs do not fall into the previously specified classes or have a single case report published in medical literature, without rechallenge [24].

According to a study by Saini et al., 141 medications have been reported to cause acute pancreatitis in the literature. However, of them, only 106 drugs were high-quality case reports [23]. For this reason, they proposed a novel classification. Saini et al. divided the drugs causing acute pancreatitis into four groups. Randomized controlled trials were included in class 1, and case–control and pharmacoepidemiologic studies were included in class 2. Class 3 were high-quality case reports that were further divided into two classes. A class 3a drug included case reports with a rechallenge and a consistent latency between drug intake and the occurrence of acute pancreatitis. Case reports with either a rechallenge or consistent latency are classified as class 3b and class 3 c. Finally, class 4 included high-quality case reports without reported latency and rechallenge. All other case reports were considered inappropriate for analysis [23]. In total, 121 medications were found to cause acute pancreatitis in the literature, according to high-quality case reports. Only three medications—6-mercaptopurine, didanosine, and azathioprine—had evidence of causing acute pancreatitis in randomized controlled clinical studies [23]. Angiotensin convertase inhibitors belong to class 2, cannabis, nitrofurantoin, trimethoprim-sulphamethoxazole to class 3b, and cyclosporine to class 3c. Tacrolimus was not mentioned in their analysis [23]. Only a few case reports of acute pancreatitis associated with the use of tacrolimus in kidney transplant patients have been reported [25,26]. 

Potential drugs implicated in the development of acute pancreatitis in our cohort include angiotensin convertase inhibitors, cannabis, tacrolimus, and sirolimus in one patient each, and simvastatin in two patients. Among numerous medications, three patients received prophylaxis with trimetoprim-sulfometoxasol within the first six months post-transplant. Rechallenge was not applied in our patients, thus disabling more precise classification of possible drug-induced pancreatitis cases. Polypharmacy is most frequently unavoidable after kidney transplantation due to the complexity of the kidney disease pathology and the maintenance of kidney allograft function. Many different medications with their possible interactions further aggravate the precise definition of the individual drug’s role in the etiology of acute pancreatitis.

Overweight and obesity are highly prevalent among kidney transplant recipients. They are often accompanied by other chronic diseases including hypertension, diabetes, and hypertriglyceridemia. Three of our patients were markedly obese, with a BMI above 30 kg/m^2^. While a BMI higher than 25 increases the risk of severe acute pancreatitis, but not mortality, meta-analysis demonstrated that a BMI > 30 raises the risk of both severity and mortality in acute pancreatitis episodes [27]. Experimental studies revealed that obesity can worsen acute pancreatitis by inducing injury to the intestinal mucosal barrier [28].

Despite being the third most common cause, hypertriglyceridemia as an inducing factor of acute pancreatitis accounts for less than 5% of all cases. However, patients who experience hypertriglyceridemia-induced pancreatitis more frequently have a severe disease course and an increased risk of persistent organ failure. Hypertriglyceridemia is typically associated with disorders of lipoprotein metabolism and is accompanied by other conditions, including diabetes, alcohol abuse, or medication use [29]. According to the literature, serum triglycerides levels should be higher than 1000 mg/dL to be considered the cause of acute pancreatitis [30,31]. None of our patients had significant hypertriglyceridemia at the time of acute pancreatitis or during the three months before its development. However, two of them were treated with simvastatin with adequate control of their lipid status. 

Despite all diagnostic attempts, etiology remains unclear in almost 30% of patients with acute pancreatitis. According to Guda et al., idiopathic acute pancreatitis is defined as pancreatitis with unknown etiology after laboratory and imaging tests [32]. Published data indicate that gallstones, microlithiasis, and sludge are the cause of idiopathic acute pancreatitis in most patients with unidentified etiology [33,34]. Finally, acute pancreatitis may be induced by obstruction of the main pancreatic or biliary duct with a benign or malignant mass [35]. For this reason, the diagnostic approach in patients with unknown etiology of acute pancreatitis should include different radiological methods including the contrast-enhanced CT scan with thin slices or MRI/magnetic retrograde cholangiopancreatography with endoscopic ultrasound examination.

A growing body of evidence demonstrated a higher risk of acute pancreatitis in patients with end-stage kidney disease. Autopsy data obtained from 78 long-term hemodialysis patients showed different pancreatic abnormalities in 60%. Acute pancreatitis was the most frequently discovered condition, occurring in 28% of cases. Additional findings included fibrotic changes, hemosiderin deposition, cysts, calcifications, signs of amyloidosis, and abscess formation [36].

The results of Rutsky et al. demonstrated that the 10-year risk of acute pancreatitis is 2.3% in patients with end-stage kidney disease [37]. Patients undergoing peritoneal dialysis are more likely than those undergoing hemodialysis to suffer from acute pancreatitis, according to this research and multiple other publications [37,38,39,40]. A possible mechanism is thought to include exposure to dialysis fluid and increased intraabdominal pressure, both leading to premature activation of proteolytic enzymes in the pancreas. Clinical presentation of acute pancreatitis in end-stage kidney disease patients treated with dialysis are the same as that in the general population. However, amylase and lipase levels might be falsely elevated in end-stage kidney disease, and for this reason, should be interpreted with caution.

The amount of data on acute pancreatitis in kidney transplant recipients is scarce. The estimated risk for the development of acute pancreatitis in this population ranges between 2.7% and 3.6% [41,42]. The relatively low rate of acute pancreatitis occurrence in our study (0.4%) compared to the literature could be explained by the large number of patients (2482) included in our multicentre, international study. Sinha et al. found 5 cases among 185 renal transplant patients in their study spanning 10 years [42], while Frick TW et al. studied a group of 224 patients with a median follow-up of 20 months [41]. The authors emphasize the unspecified etiologies of acute pancreatitis in this group of patients, including medication-associated acute pancreatitis and infectious causes, especially viral infections [42,43]. Interestingly, Chuang et al. conducted a national population-based retrospective cohort study and discovered that kidney transplant recipients have a risk of acute pancreatitis up to 3.5 times higher than the general population. Their multivariable analysis showed that there was a significant link between the risk of acute pancreatitis post kidney transplantation and alcohol consumption, gall stone disease, and a history of pancreatitis [44]. David et al. demonstrated a 68% increased risk of hypercalcemia-associated acute pancreatitis in the kidney transplant population compared to patients without kidney disease. In their study, this association did not reach statistical significance [45]. Hypercalcemia should not be underestimated, as 62% of kidney transplant patients presented with persistent hypercalcemia [46]. The risk of hypercalcemia can be decreased with calcimimetics. Hypercalcemia, which is a relatively rare cause of pancreatic injury in the general population, may be a more significant contributor to acute pancreatitis in patients with end-stage kidney disease due to its high prevalence [47]. Two of our patients had significant hypercalcemia requiring the use of cinacalcet.

Over the last few years, COVID-19 has emerged as an important contributor to acute pancreatitis-associated morbidity and mortality. However, it remains unclear whether the virus may directly damage the pancreatic cells, although both ductal and islet pancreatic cells have an abundant expression of ACE-2 receptors. In a meta-analysis of nine studies that included 3160 patients, elevated mortality rates, increased usage of mechanical ventilation and intensive care unit admission, and increased prevalence of severe pancreatitis with the need for prolonged hospitalizations for acute pancreatitis were recorded among patients diagnosed with COVID-19 when compared to those without COVID-19 [48]. They hypothesized that this fact may be the consequence of a higher degree of disease severity in patients with acute COVID-19, but also because patients have both lung damage and pronounced acute pancreatitis severity [48]. Although acute pancreatitis was recorded after vaccination against SARS-CoV-2 [49], the probability of the vaccine as the etiologic factor of acute pancreatitis is not conclusive. SARS-CoV-2 vaccine-induced pancreatitis is considered to involve different mechanisms including direct virus-mediated injury, systemic inflammatory response, circulating proinflammatory interleukins, virus-induced lipotoxicity, and drug-induced injury [50], while possible mechanisms associated with pancreatic tissue damage may include molecular mimicry, polyclonal activation of lymphocytes, activation of self-reactive lymphocytes, and vaccine-triggered release of histamine and leukotrienes [51,52]. Three patients (33%) from our cohort developed acute pancreatitis associated with acute COVID-19. We have already published our single-center experience with one patient who developed acute pancreatitis during acute COVID-19 [53]. However, in the present multicentre analysis of all acute pancreatitis cases, COVID-19 may have emerged as an important contributor to acute pancreatitis etiology after kidney transplantation. This result warrants further research. None of our patients developed acute pancreatitis after SARS-CoV-2 vaccination.

Most acute pancreatitis episodes are mild and require brief hospitalization. However, approximately 20% of patients experience more severe disease needing prolonged hospitalization or even hospitalization in an intensive care unit. Interestingly, most patients who develop a complicated course of acute pancreatitis initially present with mild disease [54]. For this reason, intensive early aggressive or moderately aggressive intravenous hydration is recommended [55], with lactated Ringer solution preferred to normal saline solution [14]. Lactated Ringer solution provides calcium [56] that binds with nonesterified fatty acids that contribute to acute pancreatitis and lactate, decreasing inflammation [57]. Antibiotics play a crucial role in treating acute pancreatitis, with infectious complications being a major contributor to morbidity and mortality in these patients. These infections include abscess formation (infected pseudocyst), infected pancreatic necrosis, cholangitis, infected fluid collections, and urinary tract infections. Antibiotics should be given if an infection is suspected but discontinued once cultures are found to be negative.

There are no formal guidelines for managing acute pancreatitis in kidney transplant recipients. Management principles are typically derived from prior experience with different groups of patients. The major therapeutic measure is aggressive hydration [13,14]. This measure may be even more important than in the general population due to the susceptibility of kidney allografts to prerenal acute kidney injury. Pain alleviation and nutritional support are important therapeutic measures [15]. Currently, no specific therapies are approved for the treatment of acute pancreatitis. Several treatments focusing on various aspects of AP development have been studied in order to advance personalized care; however, they have not been found to be effective in clinical trials [15,58,59]. Several novel medications, including anticoagulant dabigatran, monoclonal antibody infliximab, protease inhibitor ulinastatin, autophagy inhibitor spautin-A41, calcium channel inhibitor CM4620-Injectible emulsion, stem cells and nanoparticles are still under the investigation [59].

It has been emphasized that most acute pancreatitis cases are mild and self-limited. However, some patients may develop complications. Local complications include acute peripancreatic fluid collections, pseudocyst formation, and acute necrotic collections. Patients may develop splanchnic venous thrombosis and pseudoaneurysm. Systemic complications range from mild pyrexia to fatal multiorgan failure. They include circulatory shock, disseminated intravascular coagulation, respiratory insufficiency, metabolic complications (hypocalcemia, hyperglycemia, hypertriglyceridemia), pancreatic encephalopathy, retinal arteriolar obstruction, metastasis fat necrosis, and other conditions [60]. In our cohort, complications were recorded in three patients, including urinary tract infection (Morganela morganii), abscess formation, and CMV reactivation.

One of our patients developed a relapse of pancreatitis complicated by the development of an abscess in the pseudocyst. She was switched from tacrolimus to cyclosporine. The change in calcineurin inhibitor resulted in significant improvement. However, two years later, she experienced an additional episode of mild pancreatitis after the nutritional challenge. One patient who developed pancreatitis shortly after the transplantation had pseudocysts at the initial MSCT examination, indicating either earlier occurrence of pancreatitis after the transplant or even pretransplant unrecognized pancreatic injury.

Some limitations to our study should be mentioned. By being a retrospective, observational study, there is potential for selection bias. A low number of patients were identified with acute pancreatitis. Additionally, some complications may be underreported. Also, some of the patients with gastrointestinal symptoms may have had concomitant acute pancreatitis. However, laboratory and radiological analyses were not performed, and we had no precise diagnosis. Thus, number of patients with acute pancreatitis may be underestimated. Despite the limitations, this study provides valuable information on acute pancreatitis in the kidney transplant population, as this is a multicentre study performed in tertiary centers with adequate therapeutic and diagnostic options. 

## 5. Conclusions

In conclusion, while gastrointestinal symptoms are common, the diagnosis of acute pancreatitis may be delayed or even omitted in kidney transplant recipients, resulting in higher rates of morbidity and mortality. A high level of awareness and a multidisciplinary approach are essential for enhancing outcomes in this extremely vulnerable patient population.

## Figures and Tables

**Table 1:** Patient characteristics.

Patient	Age	Gender	Primary Kidney Disease	Dialysis Vintage (mo)	Type of Dialysis	Time from TX	Induction Therapy	Maintenance IS	BMI
1	48	M	CPN	10	PD	29 months	Basiliximab	Tac, MMF, S	27.3
2	53	M	Unknown	33	HD	9 years (2. tx)	Basiliximab	Tac, MMF, S	34.6
3	39	F	GN chr	45	HD/PD	24 years	None	CyA, MMF, S	26.8
4	68	F	RPGN	104	HD	16 years	Basiliximab	CyA, MMF, S	23.7
5	31	M	FSGS	17	HD	3 months	Basiliximab	Tac, MMF, S	19.2
6	33	F	ADPKD	30	PD/HD	2 months	Basiliximab	Tac, MMF, S	18.7
7	69	M	Nephroangio	38	HD	103 months	Basiliximab	Tac, MMF, S	33.1
8	69	F	CPN	54	HD	82 months	Basiliximab	CyA, MMF, S	27.5
9	52	F	GN chr	/	/	240 months	None	CyA, MMF, S	32.1
10	24	M	Unknown	/	/	21 days	ATG	Sir, MMF, S	27

TX, renal transplantation; mo, months; ATG, rabbit antithymocyteglobulin; Tac, tacrolimus; CyA, Cyclosporine A; S, Steroids; Sir, Sirolimus; MMF, Mycophenolate mofetil; M, male, F, female; HD, hemodialysis; PD, peritoneal dialysis; CPN, chronic pyelonephritis; GN chr, chronic glomerulonephritis without biopsy; tx, transplantation; BMI, body mass index; RPGN, rapidly progressive glomerulonephritis; FSGS, focal segmental glomerulosclerosis; ADPKD, autosomal dominant polycystic kidney disease; Nephroangio, nephroangiosclerosis; /, preemptive transplantation.

**Table 2:** Clinical presentation, complications, and outcome of acute pancreatitis in kidney transplant recipients.

Patient	Initial Symptoms	Possible Etiology	Amylase/Lipase (U/L)	CNI/Sir Conc	Radiological Finding	Length of Hospitalization (Days)	Complications	Rehospitalization	Outcome of AP
1	Pain, nausea	Unknown	802/2952	3.9	Parenchymal enlargement, indistinct pancreatic margins	10	None	No	Good
2	Pain	COVID, atorvastatin	505/547	6.2	Parenchymal enlargement, surrounding retroperitoneal fat stranding, enlarged lymph nodes	12	Urinart tract infection (Morganella morganii)	No	Good
3	Pain, nausea	Cholecystolithiasis	672/784	5.2	Surrounding retroperitoneal fat stranding	12 days	No	No	Good
4	Pain, nausea	COVID/CMV, simvastatin	490/491	85	Parenchymal enlargement	18	No	No	Good
5	Pain	cannabis, hypercalcemia	571/641	6.2	Hyperchogenic pancreas, indistinct pancreatic margins Pseudocyst, Surrounding retroperitoneal fat stranding	42	No	Yes	Graft loss, noncompliance
6	Pain	Unknown	731/3181	8.1	Indistinct pancreatic margins, Pseudocyst formation	34	Abscesus	Yes	Good
7	Pain	Cholecocholitiasis, hypercalcemia	Unknown	5.6	Surrounding retroperitoneal fat stranding	14	No	Yes/reccurence	Good
8	Pain, nausea	Postprocedural ERCP	2928/3636	96 ug/L	Surrounding retroperitoneal fat stranding	6	No	Yes	Good
9	Pain, nausea, vomiting	COVID	2038/3045	60.3	Surrounding retroperitoneal fat stranding, Parenchymal enlargement	22	CMV reaktiv	No	Good
10	Diarrhoea	Sirolimus	568/905	12.8	Pancreatic enlargement, indistinct margins and peripancreatic fat stranding	14	No	No	Good

CMV, cytomegalovirus; ERCP, endoscopic retrograde cholangiopancreatography.

## Data Availability

The raw data supporting the conclusions of this article will be made available by the authors upon request.

## References

[1] Gioco R., Corona D., Ekser B., Puzzo L., Inserra G., Pinto F., Schipa C., Privitera F., Veroux P., Veroux M. (2020). Gastrointestinal complications after kidney transplantation. World J. Gastroenterol..

[2] Liapis G., Boletis J., Skalioti C., Bamias G., Tsimaratou K., Patsouris E., Delladetsima I. (2013). Histological spectrum of mycophenolate mofetil-related colitis: Association with apoptosis. Histopathology.

[3] Ekberg H., Kyllönen L., Madsen S., Grave G., Solbu D., Holdaas H. (2007). Clinicians underestimate gastrointestinal symptoms and overestimate quality of life in renal transplant recipients: A multinational survey of nephrologists. Transplantation.

[4] Ponticelli C., Passerini P. (2005). Gastrointestinal complications in renal transplant recipients. Transpl. Int..

[5] Ponticelli C., Colombo D., Novara M., Basilisco G., CETRA Study Group (2010). Gastrointestinal symptoms impair quality of life in Italian renal transplant recipients but are under-recognized by physicians. Transpl. Int..

[6] Peery A.F., Crockett S.D., Murphy C.C., Lund J.L., Dellon E.S., Williams J.L., Jensen E.T., Shaheen N.J., Barritt A.S., Lieber S.R. (2019). Burden and cost of gastrointestinal, liver, and pancreatic diseases in the United States: Update 2018. Gastroenterology.

[7] Xiao A.Y., Tan M.L.Y., Wu L.M., Asrani V.M., Windsor J.A., Yadav D., Petrov M.S. (2016). Global incidence and mortality of pancreatic diseases: A systematic review, meta-analysis, and metaregression of population-based cohort studies. Lancet Gastroenterol. Hepatol..

[8] Iannuzzi J.P., King J.A., Leong J.H., Quan J., Windsor J.W., Tanyingoh D., Coward S., Forbes N., Heitman S.J., Shaheen A.A. (2022). Global incidence of acute pancreatitis is increasing over time: A systematic review and meta-analysis. Gastroenterology.

[9] Fu C.-Y., Yeh C.-N., Hsu J.-T., Jan Y.Y., Hwang T.L. (2007). Timing of mortality in severe acute pancreatitis: Experience from 643 patients. World J. Gastroenterol..

[10] Chatila A.T., Bilal M., Guturu P. (2019). Evaluation and management of acute pancreatitis. World J. Clin. Cases.

[11] Huang W., de la Iglesia-García D., Baston-Rey I., Calviño-Suarez C., Lariño-Noia J., Iglesias-Garcia J., Shi N., Zhang X., Cai W., Deng L. (2019). Exocrine pancreatic insufficiency following acute pancreatitis: Systematic review and meta-analysis. Dig. Dis. Sci..

[12] Liu J., Wang Y., Yu Y. (2020). Meta-analysis reveals an association between acute pancreatitis and the risk of pancreatic cancer. World J. Clin. Cases.

[13] de-Madaria E., Buxbaum J.L., Maisonneuve P., García de Paredes A., Zapater P., Guilabert L., Vaillo-Rocamora A., Rodríguez-Gandía M.Á., Donate-Ortega J., Lozada-Hernández E.E. (2022). Aggressive or moderate fluid resuscitation in acute pancreatitis. N. Engl. J. Med..

[14] de-Madaria E., Herrera-Marante I., Gonzalez-Camacho V., Bonjoch L., Quesada-Vázquez N., Almenta-Saavedra I., Miralles-Maciá C., Acevedo-Piedra N.G., Roger-Ibáñez M., Sánchez-Marin C. (2018). Fluid resuscitation with lactated Ringer’s solution vs normal saline in AP: A triple-blind, randomized, controlled clinical trial. United Eur. Gastroenterol. J..

[15] Sundar V., Senthil Kumar K.A., Manickam V., Ramasamy T. (2020). Current trends in pharmacological approaches for treatment and management of acute pancreatitis—A review. J. Pharm. Pharmacol..

[16] Banks P.A., Bollen T.L., Dervenis C., Gooszen H.G., Johnson C.D., Sarr M.G., Tsiotos G.G., Vege S.S., Acute Pancreatitis Classification Working Group (2013). Classification of acute pancreatitis-2012: Revision of the Atlanta classification and definitions by international consensus. Gut.

[17] Mallory A., Kern F. (1980). Drug-induced pancreatitis: A critical review. Gastroenterology.

[18] Aghdassi A.A., Weiss F.U., Mayerle J., Lerch M.M., Simon P. (2015). Genetic susceptibility factors for alcohol induced chronic pancreatitis. Pancreatology.

[19] Ammann R.W. (2001). The natural history of alcoholic chronic pancreatitis. Intern. Med..

[20] Parenti D.M., Steinberg W., Kang P. (1996). Infectious causes of acute pancreatitis. Pancreas.

[21] Meczker Á., Hanák L., Párniczky A., Szentesi A., Eross B., Hegyi P. (2020). Analysis of 1060 cases of drug-induced acute pancreatitis. Gastroenterology.

[22] Gagnon A.L., Lavoie A., Frigon M.P., Michaud-Herbst A., Tremblay K. (2020). A Drug-Induced Acute Pancreatitis Retrospective Study. Can. J. Gastroenterol. Hepatol..

[23] Saini J., Marino D., Badalov N., Vugelman M., Tenner S. (2023). Drug-Induced Acute Pancreatitis: An Evidence-Based Classification (Revised). Clin. Transl. Gastroenterol..

[24] Simons-Linares C.R., Elkhouly M.A., Salazar M.J. (2019). Drug-Induced Acute Pancreatitis in Adults: An Update. Pancreas.

[25] Xu J., Xu L., Wei X., Li X., Cai M. (2019). A case report: Acute pancreatitis associated with tacrolimus in kidney transplantation. BMC Nephrol..

[26] Liu X.H., Chen H., Tan R.Y., Luo C. (2021). Acute pancreatitis due to tacrolimus in kidney transplant and review of the literature. J. Clin. Pharm. Ther..

[27] Dobszai D., Mátrai P., Gyöngyi Z., Csupor D., Bajor J., Erőss B., Mikó A., Szakó L., Meczker Á., Hágendorn R. (2019). Body-mass index correlates with severity and mortality in acute pancreatitis: A meta-analysis. World J. Gastroenterol..

[28] Ye C., Liu L., Ma X., Tong H., Gao J., Tai Y., Huang L., Tang C., Wang R. (2019). Obesity Aggravates Acute Pancreatitis via Damaging Intestinal Mucosal Barrier and Changing Microbiota Composition in Rats. Sci. Rep..

[29] Yang A.L., McNabb-Baltar J. (2020). Hypertriglyceridemia and acute pancreatitis. Pancreatology.

[30] Toskes P.P. (1990). Hyperlipidemic pancreatitis. Gastroenterol. Clin. N. Am..

[31] Farmer R.G., Winkelman E.I., Brown H.B., Lewis L.A. (1973). Hyperlipoproteinemia and pancreatitis. Am. J. Med..

[32] Guda N.M., Muddana V., Whitcomb D.C., Levy P., Garg P., Cote G., Uc A., Varadarajulu S., Vege S.S., Chari S.T. (2018). Recurrent acute pancreatitis: International state-of-the-science conference with recommendations. Pancreas.

[33] Garg P.K., Tandon R.K., Madan K. (2007). Is biliary microlithiasis a significant cause of idiopathic recurrent AP? A long-term follow-up study. Clin. Gastroenterol. Hepatol..

[34] Lee A., Ko C., Buitrago C., Hiramoto B., Hilson L., Buxbaum J., NS-LR Study Group (2021). Lactated ringers vs normal saline resuscitation for mild acute pancreatitis: A randomized trial. Gastroenterology.

[35] Tandon M., Topazian M. (2001). Endoscopic ultrasound in idiopathic acute pancreatitis. Am. J. Gastroenterol..

[36] Vaziri N.D., Dure-Smith B., Miller R., Mirahmadi M. (1987). Pancreatic pathology in chronic dialysis patients—An autopsy study of 78 cases. Nephron.

[37] Rutsky E.A., Robards M., Van Dyke J.A., Rostand S.G. (1986). Acute pancreatitis in patients with end-stage renal disease without transplantation. Arch. Intern. Med..

[38] Bruno M.J., van Westerloo D.J., van Dorp W.T., Dekker W., Ferwerda J., Tytgat G.N., Schut N.H. (2000). Acute pancreatitis in peritoneal dialysis and haemodialysis: Risk, clinical course, outcome, and possible aetiology. Gut.

[39] Lankisch P.G., Weber-Dany B., Maisonneuve P., Lowenfels A.B. (2008). Frequency and severity of acute pancreatitis in chronic dialysis patients. Nephrol. Dial. Transplant..

[40] Quraishi E.R., Goel S., Gupta M., Catanzaro A., Zasuwa G., Divine G. (2005). Acute pancreatitis in patients on chronic peritoneal dialysis: An increased risk?. Am. J. Gastroenterol..

[41] Frick T.W., Fryd D.S., Sutherland D.E., Goodale R.L., Simmons R.L., Najarian J.S. (1987). Hypercalcemia associated with pancreatitis and hyperamylasemia in renal transplant recipients: Data from the Minnesota randomized trial of cyclosporine versus antilymphoblast azathioprine. Am. J. Surg..

[42] Sinha S., Jha R., Lakhtakia S., Narayan G. (2003). Acute pancreatitis following kidney transplantation—Role of viral infections. Clin. Transplant..

[43] Foitzik T., Forgacs B., Ryschich E., Hotz H., Gebhardt M.M., Buhr H.J., Klar E. (1998). Effect of different immunosuppressive agents on acute pancreatitis: A comparative study in an improved animal model. Transplantation.

[44] Chuang Y.W., Huang S.T., Yu T.M., Li C.Y., Chung M.C., Lin C.L., Chang C.S., Wu M.J., Kao C.H. (2019). Acute pancreatitis risk after kidney transplantation: Propensity score matching analysis of a national cohort. PLoS ONE.

[45] David D.S., Sakai S., Brennan B.L., Riggio R.A., Cheigh J., Stenzel K.H., Rubin A.L., Sherwood L.M. (1973). Hypercalcemia after renal transplantation—Long-term follow-up data. N. Engl. J. Med..

[46] Ramirez-Sandoval J.C., Marino L., Cojuc-Konigsberg G., Reul-Linares E., Pichardo-Cabrera N.D., Cruz C., Hernández-Paredes E.N., Berman-Parks N., Vidal-Ruíz V., Estrada-Linares J.M. (2023). Long-term effects of hypercalcemia in kidney transplant recipients with persistent hyperparathyroidism. J. Nephrol..

[47] Seng J.J.B., Tan Y.L.C., Lim R.W., Ng H.T.S., Lee P.H., Wong J. (2018). Prevalence and risk factors for hypercalcemia among non-dialysis patients with chronic kidney disease-mineral and bone disorder. Int. Urol. Nephrol..

[48] Zhu C., Wu H., Yang X., Gao J. (2024). The outcomes of COVID-19 and acute pancreatitis: A systematic review and meta-analysis. Transl. Gastroenterol. Hepatol..

[49] Cahuapaza-Gutierrez N.L., Pajuelo-Vasquez R., Quiroz-Narvaez C., Rioja-Torres F., Quispe-Andahua M., Runzer-Colmenares F.M. (2024). Acute abdomen following COVID-19 vaccination: A systematic review. Clin. Exp. Vaccine Res..

[50] Correia de Sá T., Soares C., Rocha M. (2021). Acute pancreatitis and COVID-19: A literature review. World J. Gastrointest. Surg..

[51] Bogdanos D.P., Smith H., Ma Y., Baum H., Mieli-Vergani G., Vergani D. (2005). A study of molecular mimicry and immunological cross-reactivity between hepatitis B surface antigen and myelin mimics. Clin. Dev. Immunol..

[52] Stöllberger C., Kastrati K., Dejaco C., Scharitzer M., Finsterer J., Bugingo P., Melichart-Kotik M., Wilfing A. (2023). Necrotizing pancreatitis, microangiopathic hemolytic anemia and thrombocytopenia following the second dose of Pfizer/BioNTech COVID-19 mRNA vaccine. Wien. Klin. Wochenschr..

[53] Basic-Jukic N., Juric I., Katalinic L., Furic-Cunko V., Sesa V., Mrzljak A. (2024). Acute pancreatitis as a complication of acute COVID-19 in kidney transplant recipients. World J. Clin. Cases.

[54] Banks P.A., Freeman M.L. (2006). Practice Parameters Committee of the American College of Gastroenterology. Practice guidelines in acute pancreatitis. Am. J. Gastroenterol..

[55] Gardner T.B. (2022). Fluid resuscitation in AP: Going over the waterfall. N. Engl. J. Med..

[56] Khatua B., Yaron J.R., El-Kurdi B., Kostenko S., Papachristou G.I., Singh V.P. (2020). Ringer’s lactate prevents early organ failure by providing extracellular calcium. J. Clin. Med..

[57] Hoque R., Farooq A., Ghani A., Gorelick F., Mehal W.Z. (2014). Lactate reduces liver and pancreatic injury in Toll-like receptor and inflammasome-mediated inflammation via GPR81-mediated suppression of innate immunity. Gastroenterology.

[58] Garami A., Hegyi P. (2023). Precision Medicine in Pancreatitis: The Future of Acute Pancreatitis Care. Function.

[59] Hey-Hadavi J., Velisetty P., Mhatre S. (2023). Trends and recent developments in pharmacotherapy of acute pancreatitis. Postgrad. Med..

[60] Johnson C.D., Besselink M.G., Carter R. (2014). Acute pancreatitis. BMJ.

